# Co‐creating practical social media recommendations for dementia prevention researchers: A Delphi study

**DOI:** 10.1002/alz.085453

**Published:** 2025-01-09

**Authors:** Viorica Hrincu, Katie T. Roy, Julie M. Robillard

**Affiliations:** ^1^ University of British Columbia, Vancouver, BC Canada; ^2^ BC Children’s and Women’s Hospital, Vancouver, BC Canada

## Abstract

**Background:**

Social media provides dementia prevention researchers with additional opportunities to engage diverse audiences, including healthy individuals who unaware of their eligibility to take part in dementia‐related research. However, practical social media guidance that reflects the values and priorities of potential participants is needed. To address this gap, we sought to create consensus recommendations with research professionals and community experts.

**Method:**

We conducted a three‐round, modified Delphi consisting of three online surveys and three conferences calls. Based on data from earlier project phases, a group of 16 experts with lived (n = 10) and professional (n = 6) experiences co‐created a set of recommendations to guide ethical social media use for dementia prevention research. Consensus was defined *a priori* as ≥70% agreement.

**Result:**

Twenty‐six items attained panelist agreement. Two privacy‐related items reached consensus in Round 1: the ethical appropriateness of closed social media groups (88%) and accessing individuals not on social media through social media contacts (79%). Nine items reached consensus in Round 2, including addressing misinformation (79%), stigma (93%), and other pertinent items for social media communication (e.g., public criticism, explaining process of science). Fifteen of the sixteen remaining items reached consensus after revision in Round 3. These items included defining appropriate comments (100%), rules of engagement on dementia social media pages (100%, e.g., positive messaging, relevant topics), and ranking appropriate prevention language use for different audiences (e.g., young, healthy adults, individuals with a family history). One item pertaining to language use for people living with dementia did not reach consensus.

Recommendations were organized into seven social media use cases: setting up a social media page, handling online misinformation, actively challenging stigma, handling difficult online interactions, introducing new research to the public, help with study recruitment, and the language of prevention when writing posts.

**Conclusion:**

Research professionals and community members co‐created consensus recommendations to facilitate the ethical use of social media by dementia prevention researchers. By providing practical guidance, these recommendations will uphold ethical decision‐making on social media. Next steps are to create an evaluation tool and distribute the recommendations to relevant audiences.

